# Finished Genome Sequence of the Highly Multidrug-Resistant Human Urine Isolate *Citrobacter freundii* Strain SL151

**DOI:** 10.1128/genomeA.01225-16

**Published:** 2016-11-03

**Authors:** Tomasz A. Leski, Chris R. Taitt, Umaru Bangura, Rashid Ansumana, David A. Stenger, Zheng Wang, Gary J. Vora

**Affiliations:** a Center for Bio/Molecular Science and Engineering, U.S. Naval Research Laboratory, Washington, District of Columbia, USA; b Mercy Hospital Research Laboratory, Bo, Sierra Leone; c Department of Community Health Sciences and Clinical Studies, Njala University, Bo, Sierra Leone

## Abstract

*Citrobacter freundii* is a Gram-negative opportunistic pathogen that is increasingly being recognized as a causative agent of hospital-acquired urinary tract infections and an important reservoir of antimicrobial resistance determinants. In this report, we describe the finished genome sequence of *C. freundii* strain SL151, a highly multidrug-resistant human urine isolate.

## GENOME ANNOUNCEMENT

*Citrobacter freundii* is recognized as an emerging opportunistic pathogen and is known to cause a variety of ailments (e.g., urinary tract infections [UTIs], wound infections, gastrointestinal infections, septicemia, meningitis), especially in immunocompromised patients and in hospital settings (1–5). This emergence has coincided with the finding that *C. freundii* is often resistant to multiple classes of antibiotics, suggesting that both clinical and environmental strains may be important reservoirs of antimicrobial resistance determinants (ARDs) (6–10). For example, a recent survey of outpatients in Bo, Sierra Leone, revealed that a surprisingly high number of *C. freundii* were isolated from the urine of individuals with UTI symptoms and that all of these isolates were highly multidrug-resistant (MDR) (11). To determine the underlying genetics responsible for these observed MDR phenotypes, we sequenced the genome of a representative isolate from this collection, *C. freundii* strain SL151, using the Pacific Biosciences RS II sequencing platform (DNA Link USA, Inc., San Diego, CA, USA).

Genomic DNA was extracted using the Gentra Puregene yeast/bacteria Kit (Qiagen, Valencia, CA, USA) and used to prepare a 20-kb insert library that was sequenced using a single-molecule real-time (SMRT) sequencing cell and P6-C4 chemistry. This resulted in 7,148 filtered and preassembled sequence reads with a mean length of 17,444 bp and 23× genome coverage. Assembly and consensus polishing (via SMRT Analysis version 2.3.0 and HGAP.2) yielded one circular chromosome (5,073,255 bp, 51.7% GC) and two circular plasmids (210,673 bp, 54.7% GC, and 154,967 bp, 53.7% GC) with a finished genome size of 5,438,895 bp. Gene prediction and annotation were performed using GeneMarkS+ and the NCBI Prokaryotic Genome Annotation Pipeline, respectively, and identified 5,537 coding sequences, of which 1,127 were predicted to encode hypothetical proteins.

A standard BLAST query of the Comprehensive Antibiotic Resistance Database (CARD) (12) using the SL151 genome sequence resulted in the identification of 97 resistance-associated genes, of which 36 demonstrated ≥95% nucleotide identity to the CARD reference sequences and provided the underlying genotype for every observed resistance phenotype. ARDs found on the chromosome include the intrinsic *C. freundii* AmpC cephalosporinase encoded by the *bla*_CMY-79_ gene, a truncated copy of a *qnrB* gene, and a number of multidrug efflux pump–encoding genes. The larger plasmid contained the *aac(3)-III*, *bla*_TEM-1_, and *bla*_CTX-M-15_ genes in a Tn2-like structure identical to *Klebsiella pneumoniae* pENVA (13), as well as the *qnrS1*, *sul2*, *catA1*, *strA*, *strB*, and *aadA16* genes. Finally, the smaller plasmid was found to contain an ~11-kb-long, ARD-rich, composite class 1 integron harboring *aac(6*′*)-Ib-cr*, *arr*-3, *dfrA27*, *aadA16*, and *qnrB6* gene cassettes. This composite element is nearly identical to structures found in *K. pneumoniae* and *K. oxytoca* plasmids (14, 15). Interestingly, a mutated duplication of this region was found adjacent to the fully functional copy. Also detected was an IS*26*-flanked composite transposon containing the *catA2* and *tetD* genes that is identical to a locus from the *Salmonella* sp. plasmid pTC67. Overall, this genome highlights the mosaic plasmid-mediated accumulation of ARDs in *C. freundii*, particularly those ARDs conferring resistance to aminoglycoside, fluoroquinolone, and sulfonamide compounds.

### Accession number(s).

This whole-genome project has been deposited at DDBJ/EMBL/GenBank under the accession numbers CP016952, CP017058, and CP017059.
